# Chiral‐at‐Metal Racemization of Tris‐Chelated Octahedral Complexes: A DFT Case Study of Al(acnac)_3_

**DOI:** 10.1002/chem.71610

**Published:** 2026-08-21

**Authors:** George Dhimba, Alfred Muller, Koop Lammertsma

**Affiliations:** ^1^ Department of Chemical Sciences University of Johannesburg Johannesburg South Africa; ^2^ Department of Chemistry and Pharmaceutical Sciences, Faculty of Sciences Vrije Universiteit Amsterdam Amsterdam The Netherlands

**Keywords:** aluminum, coordination modes, density functional calculations, N,O‐ligands, racemization

## Abstract

Configurationally stable chiral M(α‐chel)_3_ complexes (α‐chel = asymmetric chelating ligand) play a central role in asymmetric catalysis, yet the fundamental processes governing their racemization remain poorly understood. Density functional theory calculations at the ωB97X‐D/def2tzvpp and ωB97X‐D/6‐311++G(d,p) levels were employed to investigate the racemization and isomerization mechanisms of Al(acnac)_3_ as a prototypical M(α‐chel)_3_ system. Of the *mer* and *fac* enantiomeric isomers the *mer* form is favored by 1.7 kcal mol^−1^. Their isomerization is kinetically challenging with a 26.7 kcal mol^−1^ high‐energy *d,l‐cis* pathway. Racemization of *mer*‐Al(acnac)_3_ via either *trans* or *d,l‐trans* transition structures occur with slightly lower barriers of approximately 20 kcal mol^−1^ for both. Methyl substitution of all N‐chelate sites enhances the stabilization of the *mer* isomer over the *fac* isomer to 5.8 kcal mol^−1^ and inhibits access to the *cis*, *trans*, and *d,l‐cis* transition structures due to steric repulsion. This leaves the *d,l‐trans* pathway as the sole channel for Δ‐*mer* ⇌ Λ‐*mer* racemization, albeit with a very substantial barrier of 28.1 kcal mol^−1^. These results establish a unified mechanistic and topological framework for racemization in M(α‐chel)_3_ complexes and provide design principles for developing robust chiral‐at‐metal architectures.

## Graphical abstract

**Racemization and isomerization** of M(a‐chel)_3_ complexes was unraveled using Al(acnac)_3_ as a model. The ∆/Λ‐*fac* isomers have four racemization paths of which one includes isomerization to and racemization of the *mer* isomer. N‐substitution should enable separation of the ∆‐*fac* and Λ‐*fac* enantiomers.

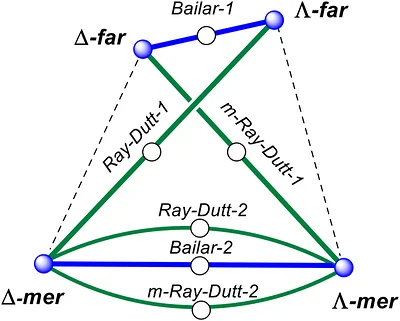

## 1 Introduction

Octahedral tris‐chelate complexes of the general formula M(chel)_3_, where chel is a bidentate ligand, represent a cornerstone in coordination chemistry. They have served for decades as model systems for understanding metal ligand bonding, stereochemistry, and intramolecular dynamics [1, 2, 3]. When three bidentate ligands coordinate to an octahedral metal center, the resulting arrangement is inherently chiral, giving rise to an enantiomeric Δ and Λ pair. The configurational stability of the enantiomers and the mechanisms by which they interconvert have been of experimental and theoretical interest owing to their implications in asymmetric catalysis [3, 4, 5, 6].

Racemization of tris‐chelate complexes proceeds via intramolecular rearrangements that preserve the metal coordination number and occur without ligand dissociation. The two nondissociative processes distinguished by the symmetry axis of rotation are the Bailar twist [7], which involves rotation of ligands through a D_3h_ trigonal prismatic transition structure, and the Ray–Dutt twist [8], which proceeds via a lower‐symmetry C_2v_ transition structure. These mechanisms have been addressed experimentally [9, 10, 11, 12, 13, 14] and computationally [15, 16, 17] for octahedral tris‐chelate complexes.

When the chelates are asymmetric (a‐chel), such as β‐diketoiminate (acnac), the number of possible minimum energy structures doubles [18, 19, 20, 21]. Rather than just a single Δ and Λ pair, the complex exists as two pairs of enantiomers: *trans meridional* (abbreviated as *mer*, where two identical donor atoms are in *trans* positions) and *cis* or *facial* (abbreviated as *fac*, where three identical donor atoms occupy the same triangular face) as shown in Figure 1. This adds a layer of complexity to the racemization process as also the number of twisting transition structures doubles.

**FIGURE 1:**
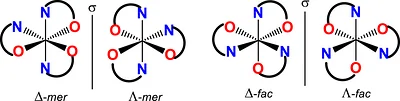
Schematic illustration of enantiomeric Δ and Λ forms of *meridional* (*mer*) and *facial* (*fac*) isomers of M(acnac)_3_.

Over half a century ago, Muetterties provided topological graphs for the rearrangements of both octahedral bis‐chelate and tris‐chelate complexes [22]. Recently, we reported on the behavior of molybdenum dioxo bis‐chelate systems with acetylacetonate (acac) [23], β‐diketoiminate (acnac) [24], and β‐diketiminate (nacnac) [25] as well as on the tungsten analogues [26]. Their Δ/Λ‐racemization was shown to be feasible by four pathways, that is, the established Bailar and Ray–Dutt twists and the more recently recognized Conte–Hippler (CH) [27] and Dhimba–Muller–Lammertsma (DML) twists. Racemization of these complexes with symmetrical ligands (acac, nacnac) fully complies with Muetterties topological graph, which is, however, more complex with an asymmetric ligand (acnac). The racemization was shown to become severely hindered by N‐substitution of the acnac and nacnac ligands [25]. In the present study we address the behavior of the tris‐chelated aluminum complex with the asymmetric acnac ligand, which is subject to only the Bailar and Ray–Dutt twists for molecular symmetry reasons.

Muetterties topological graph for the isomerization/racemization of tris‐chelate complexes with asymmetric ligands is given in Figure 2 that also shows the Bailar and Ray–Dutt twists using Muetterties’ nomenclature [22]. For over half a century the applicability of such metal complexes has received only modest attention. Systems with transition metals, like U(acnac)_3_ [28], Ru(acnac)_3_ [29, 30], and Ru(acnac)_2_(CO)_2_ [31] have been studied with a focus on structural data. Other reported tris(a‐chel)_3_ metal complexes include V(L‐glycinato)_3_ [32], *mer*‐Fe(phenol‐oxazoline)_3_ [33] Ga(l‐phenyl‐5‐methylhexane‐2,4‐dionato)_3_ [9], and the tris α‐isopropenyl‐ and α‐isopropyltropolonato complexes of Al, Co, Ga, Ge, Mn, Rh, Ru, and V [12] of which the Rh and Ru complexes are rigid in contrast to the others, but with limited mechanistic detail.

**FIGURE 2:**
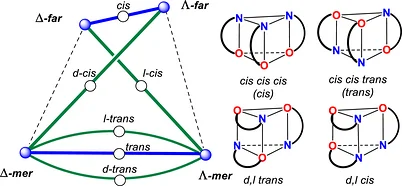
Topological map of racemization in octahedral M(a‐chel)_3_ complexes. Enantiomeric pairs Δ‐*mer*/Λ‐*mer* and Δ‐*fac*/Λ‐*fac* occupy the vertices of the tetrahedron (filled circles), which are interconnected by transition structures represented as open circles. Blue lines represent the Bailar twists and the green lines the Ray–Dutt twists. Dashed imaginary lines are used to complete the tetrahedron.

Racemization of tris‐chelate octahedral complexes can be addressed experimentally with variable‐temperature (VT) NMR, dynamic NMR (DNMR), circular dichroism (CD), reaction kinetics, and chiral chromatographic techniques, among others. Saalfrank and coworkers [34] monitored the enantiomerization of homochiral octahedral magnesium clusters by VT‐NMR and concluded that stereochemical inversion proceeds through simultaneous Bailar twists at four magnesium centers. Likewise, Raymond and coworkers [35] investigated chiral inversion and isomerization mechanisms in gallium(III) catecholate complexes by kinetic methods and concluded that inversion mechanisms proceeded through a nondissociative trigonal‐prismatic transition state (e.g., Bailar or Ray–Dutt twists) rather than via bond rupture. Computational studies can, of course, provide detail on the twisting mechanisms that govern the configurational stability of octahedral tris‐chelate complexes.

Aluminum has been studied for its coordination chemistry and applicability in homogeneous catalysis [36, 37]. Here, we address the racemization and isomerization of Al(acnac)_3_ and strategies to suppress these processes. It serves as representative model for the general tris‐chelate M(α‐chel)_3_ system in the pursuit of designing racemization‐resistant octahedral complexes for chiral‐at‐metal catalysis. The presumed fast Δ ⇌ Λ racemization of tris‐chelate complexes (e.g., acac [38], and α‐isopropenyl‐ and α‐isopropyl‐tropolonato [12]) render them suitable to explore racemization and isomerization pathways by computational means; the methyl substituted variant Al(acnac^Me3^)_3_ is known as a precursor in chemical vapor deposition [39].

Whereas chiral‐at‐metal complexes used in asymmetric catalysis are mostly of the bis‐chelate family, the tris‐chelate complexes are relevant. For example, (i) enantiopure Λ‐Cr(acac)_3_ was used as a chiral initiator in enantioselective Soai reactions [40], and the D_3_‐symmetrical cobalt (III) trications derived from Werner's seminal work, such as [Co(en)_3_]^3+^ and its derivatives, can induce chirality in, for example, α‐aminations of 1,3‐dicarbonyl compounds [41], ring‐opening polymerizations of lactide [42], and Michael additions [43, 44] among others.

## 2 Computational Details

All density functional theory (DFT) calculations were executed with the Gaussian 16 [45] ver. C01 program suite. Geometry optimizations for the Al(acnac)_3_ *mer* and *fac* isomers and the Bailar and Ray–Dutt transition structures associated with racemization, were performed using the range‐separated hybrid functional ωB97X‐D [46]. Two basis sets (6‐311++G(d,p) [47, 48, 49] and def2tzvpp) [50] were employed and vibrational frequency analyses [45] were performed at the same level of theory as the geometry optimizations to characterize all stationary points on the potential energy surface. The def2tzvp basis set provides triple‐ζ valence description with polarization functions for all elements from H to Rn and has been benchmarked for DFT calculations on both main‐group and transition‐metal systems [50]. Throughout, the reported structures and energies are from the def2tzvpp basis set, with energies calculated at the 6–311++G(d,p) level given in parentheses. Optimized minima were confirmed by the absence of imaginary frequencies, while transition structures exhibited a single imaginary frequency corresponding to the expected racemization coordinate, associated with either Bailar or Ray–Dutt twist motions. Intrinsic reaction coordinate (IRC) [51, 52, 53] calculations were conducted to verify the connectivity of each transition structure with the corresponding enantiomeric minima. To reduce computational cost, IRC calculations were performed at a lower ωB97X‐D/6‐31G(d,p) level. The resulting IRC pathways were followed in both forward and reverse directions and confirmed to smoothly connect the relevant Δ/Λ *mer* or *fac* isomers.

## 3 Results and Discussion

### 3.1 Geometries of *mer* and *fac* Al(acnac)_3_ isomers

The calculated Δ/Λ‐*mer* and Δ/Λ‐*fac* geometries of Al(acnac)_3_ are shown in Figure 3 and their energies are given in Table 1. The relative energies obtained with the 6–311++G(d,p) and def2TZVP basis sets are very similar giving confidence in the methodology used. Both *mer* and *fac* enantiomers adopt octahedral geometries, with the *mer* isomer marginally favored by 1.7 *(1.7)* kcal mol^−^^1^. Each acnac chelate exhibits a ca. 88° bite angle at the aluminum center for both the *mer* and *fac* enantiomers. The pseudo‐trans angles ∠_(trans)_ at aluminum for the *mer* isomer are 174.4° for O–Al–O_(trans)_ and 177.0° for N–Al–N_(trans)_, while mixed N–Al–O_(trans)_ display an angle of 176.4°. In the *fac* isomer, all three trans N–Al–O_(trans)_ angles are equivalent at 177.4°, while the O–Al–O_(cis)_, and N–Al–N_(cis)_, angles are 90.2° and 89.6°, respectively. Al–O_(trans)_ bonds are 1.907 and 1.894 Å, while Al–N_(trans)_ distances are 1.983 and 1.877 Å for the *mer* isomer. Similarly, Al–O_(cis)_ and Al–N_(cis)_ bonds in the *mer* isomer are 1.883 and 1.997 Å, respectively. In contrast, all three Al─N and Al─O bonds in the *fac* isomer are approximately equal with bond lengths of 1.995 and 1.886 Å, respectively.

**FIGURE 3:**
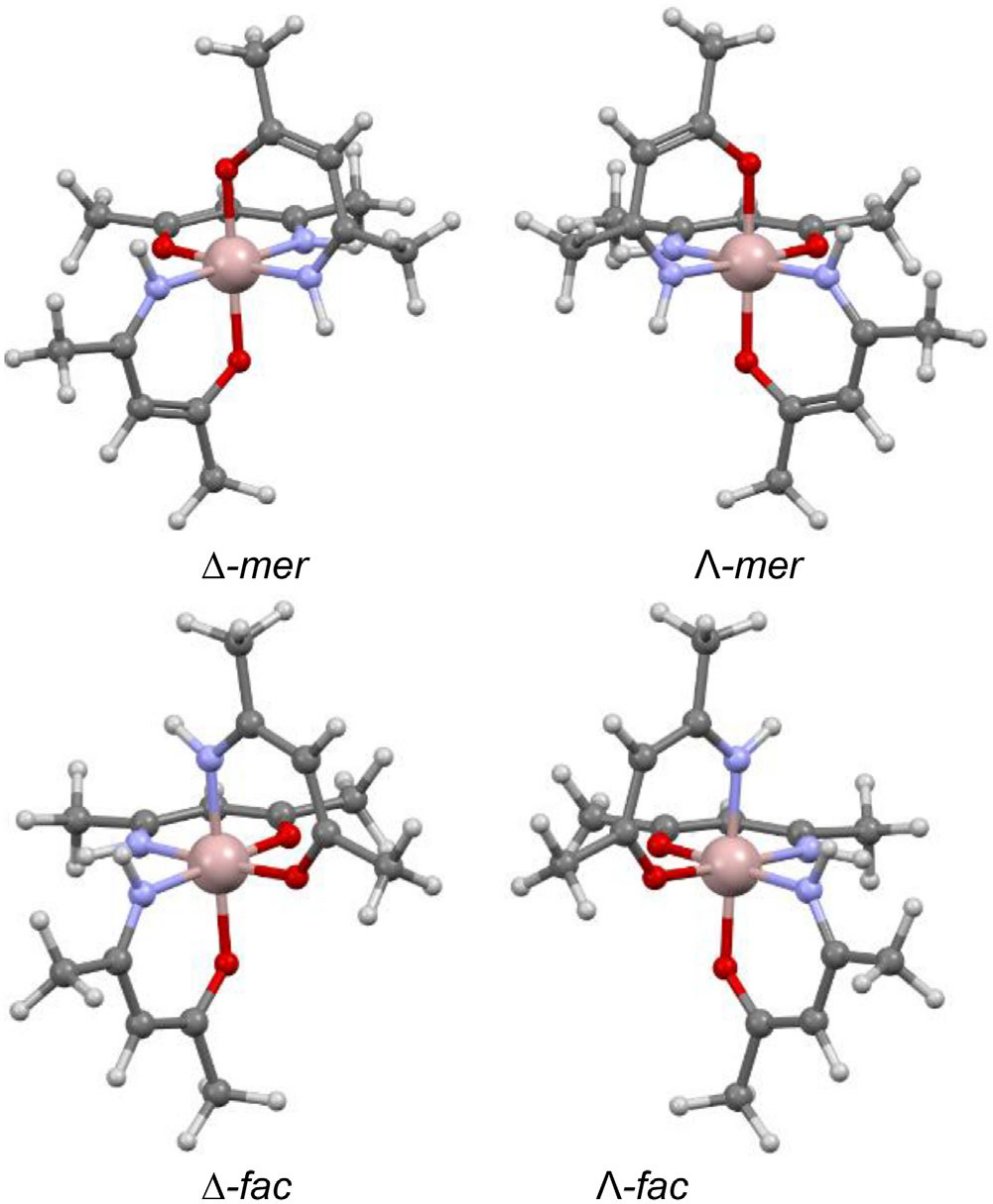
Geometry optimized molecular structures of the *mer‐* and *fac*‐enantiomers of Al(acnac)_3_.

**TABLE 1:** Relative energies (∆*E*) of the *mer* and *fac* enantiomers, and the transition structures of Al(acnac)_3_.

Structure	Twist	def2tzvpp	6‐311++G(d,p)
Δ/Λ‐*mer*		0.0	0.0
Δ/Λ‐*fac*		1.7	1.7
*cis*	Bailar	29.0	29.6
*trans*	Bailar	19.8	19.4
*d/l‐cis*	Ray–Dutt	26.7	27.1
*d/l‐trans*	Ray–Dutt	20.2	19.9

### 3.2 The Bailar Twists

The two Bailar twist transition structures, designated as *cis* and *trans* in the topological graph shown in blue color in Figure 2, are depicted in Figure 4; for historic, legibility, and simplicity reasons we use Muetterties original nomenclature throughout as the formal IUPAC nomenclature is cumbersome to use [54, 55]. The *cis* structure, like the *fac* isomer, has the three identical N or O atoms on the same side, whereas the *trans* structure resembles the *mer* isomer. In this context, the *trans* Bailar twist reflects racemization of the Δ and Λ *mer* enantiomers, whereas the *cis* form corresponds to racemization of the *fac* enantiomers. Notably, the *cis* structure possesses *C_3_* symmetry, such that the oxygen atoms may be positioned directly behind the nitrogen atoms, and vice versa. By contrast, the *trans* structure exhibits the lower *C_s_* symmetry.

**FIGURE 4:**
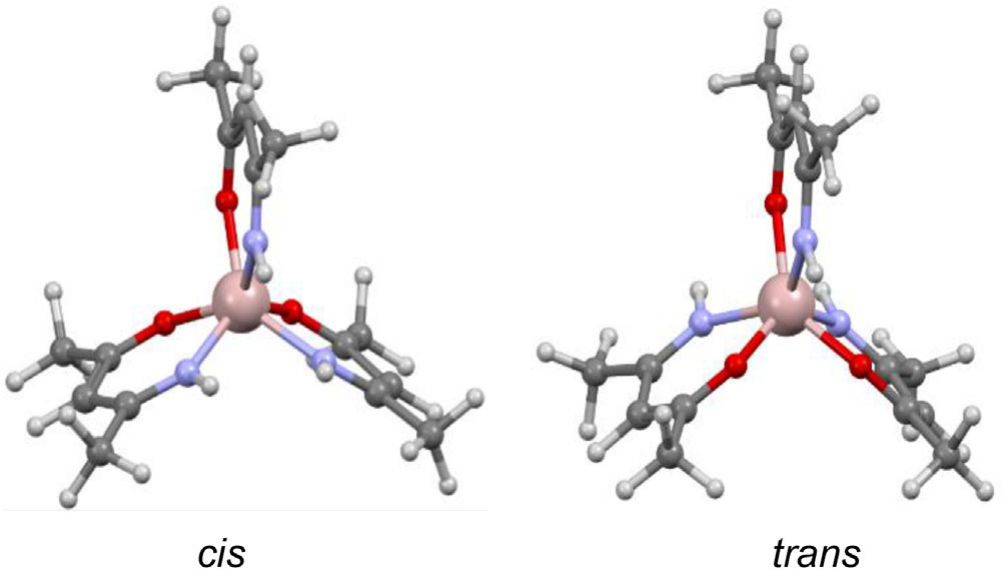
Molecular structures of the two Bailar twists, designated *cis* and *trans* according to the orientation of their chelate ligands.

Relative to the *fac* isomer, the Al─O and Al─N bond lengths in the *cis* twist are both elongated and inequivalent. Their Al─O and Al–N distances are 1.912, 1.913, 1.909 Å and 2.050, 2.054, 2.048 Å, respectively. Similarly, the Al─O and Al─N bonds in the *trans* twist are also longer than those in the *mer* minimum, with the two Al–O and two Al–N bonds on the same side of aluminum being equivalent with lengths of 1.951 and 2.001 Å, respectively. The remaining Al─O and Al─N bonds of the *trans* twist structure are 2.000 and 1.962 Å long, respectively.

The energy difference between the *cis* transition structure and the global *mer* minimum amounts to a significant 29.0 *(29.6)* kcal mol^−1^. The *cis* structure is notably also less stable than the *trans* form by 9.2 *(10.2)* kcal mol^−1^, the origin of which can be rationalized on comparing the presence of H···H contacts in the two Bailar structures. In the *cis* form, three such contacts are observed at 2.280 (×2) and 2.288 Å, all of which are shorter than the sum of the van der Waals radii for hydrogen. By contrast, the *trans* structure exhibits a single H···H contact of 2.452 Å, which is slightly longer than the sum of the van der Waals radii. Thus, increased steric repulsion in the *cis* transition structure likely contributes to its reduced stability.

### 3.3 The Ray–Dutt Twists

The two enantiomeric Ray–Dutt twists pairs are depicted schematically in Figure 5. Both the *d‐trans* and *l‐trans* transition structures connect the Δ‐*mer* and Λ‐*mer* enantiomers [42]. In sharp contrast, the *d,l‐cis* pair represents instead isomerization that involves inversion of configuration, thereby connecting Δ‐*mer* with the Λ‐*fac* enantiomer (*d‐cis)* and Λ‐*mer* with the Δ‐*fac* enantiomer (*l‐cis*) as shown by the green colored lines in the Graph in Figure 2. Racemization of the Δ/Λ‐*mer* enantiomers by means of the d*,l‐trans* pathways requires 20.2 *(19.9)* kcal mol^−1^ while the *d,l‐cis* isomerization pathways are less favored with an energy difference of 26.7 (*27.1*) kcal mol^−^^1^. Similar to the noted two Bailar twists, the energy difference of 6.5 (*7.2*) kcal mol^−^^1^ between the two Ray–Dutt twists arises from the distinct orientation of the three acnac chelates (Figure 6).

**FIGURE 5:**
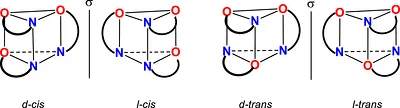
Schematic representation of the two pairs of enantiomeric Ray–Dutt transition structures for racemizing Al(acnac)_3_.

**FIGURE 6:**
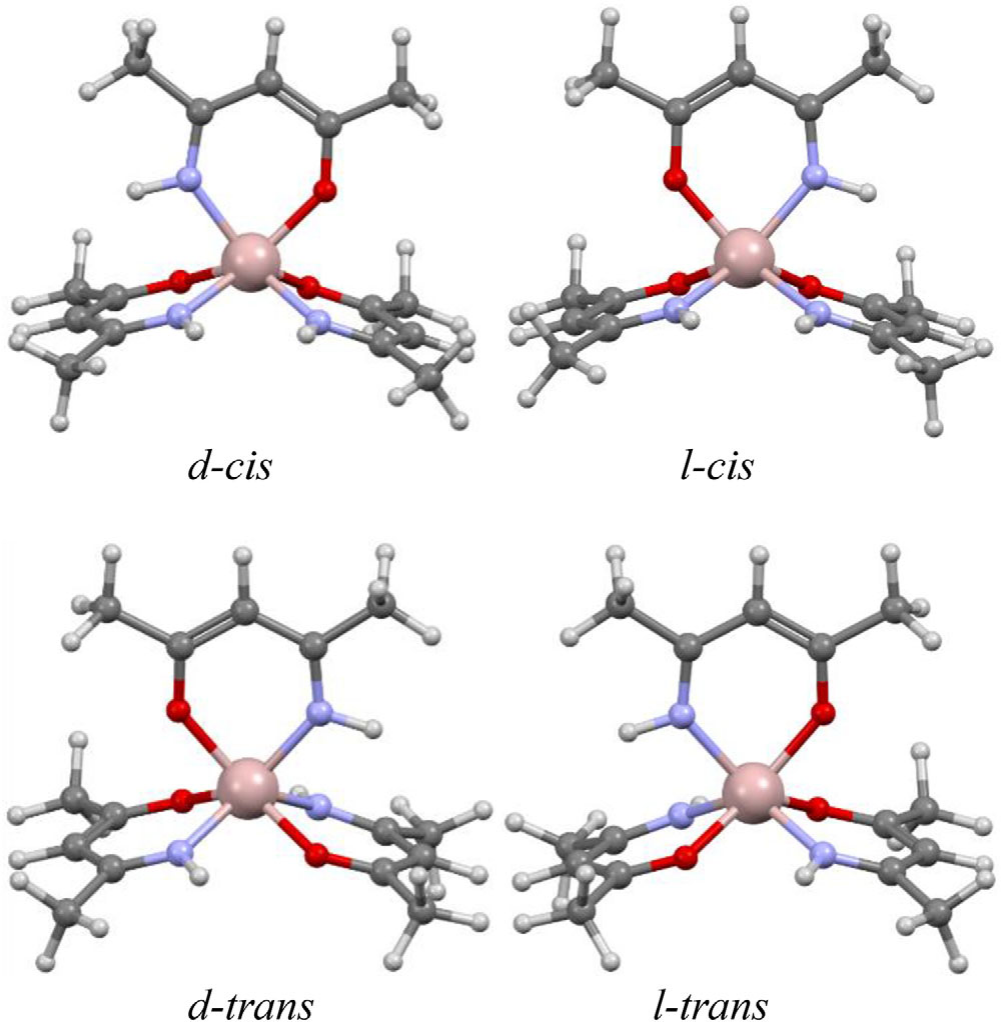
Molecular structures for the *d,l‐cis* (top) and *d,l‐trans* (bottom) Ray–Dutt twists for Al(acnac)_3_.

In the less favored *d,l*‐*cis* structures, two donor N atoms (as well as the O atoms) from adjacent acnac chelates, whose ligand planes deviate from planarity by 31.7°, are on the same side of the Al coordination sphere. As a consequence, the two N‐bound H atoms are in close contact with an H···H separation of 2.100 Å. This distance is shorter than the sum of the van der Waals radii of two hydrogen atoms indicating the presence of steric H···H destabilization. In contrast, in the *d,l*‐*trans* transition structure the corresponding H atoms associated with the two near‐planar acnac chelates (28.4° deviation from planarity) are positioned on opposite sides of the Al coordination sphere and are therefore devoid of an H···H interaction. This spatial arrangement minimizes steric repulsion and stabilizes the *d,l*‐trans transition structure relative to its *d,l*‐cis analogue. Both *d,l*‐cis and *d,l*‐trans transition structures possess *C_1_* point‐group symmetry in contrast to the higher *C_3_* and *C_s_* symmetries found for the Bailar twists.

### 3.4 Racemization of Al(acnac)_3_

Racemization and isomerization of Al(acnac)_3_ are well represented by Muetterties’ topological tetrahedron shown in Figure 2. The global minimum is the *mer* isomer which has four racemization pathways connecting its Δ and Λ enantiomers, three of which involve a single step, namely the enantiomeric *d‐trans* and *l‐trans* twists (Ray–Dutt) and the *trans* twist (Bailar). The fourth pathway is a three‐step process that starts by isomerizing a *mer* enantiomer to a *fac* enantiomer (e.g., *d‐cis* (Ray–Dutt)), converting it to the other *fac* enantiomer (*cis* (Bailar)), and then isomerizing (*l‐cis* (Ray–Dutt)) to the other *mer* enantiomer. For this process, racemization of the *fac* isomer is the rate determining step (29.0 (*29.6*) kcal mol^−1^), but the energy difference of 2.3 (*2.5*) kcal mol^−1^ with *mer‐fac* isomerization barrier is modest. In contrast, the three direct racemization pathways for *mer*‐Al(acnac)_3_ are much more favorable with the *trans* twist (Bailar) of 19.8 (*19.4*) kcal mol^−1^ that is with 0.4 (*0.5*) kcal mol^−1^ barely preferred over the enantiomeric *d,l‐trans* twists (Ray–Dutt).

### 3.5 *N*‐Methyl Substitution of Al(Acnac)_3_

Can Al(acnac)_3_ be modified to the extent that one enantiomer could have chiral integrity? Accomplishing such a feat requires all four racemization pathways to have insurmountable nondissociative barriers. The obvious way to pursue such an approach is to substitute the N‐sites of the acnac ligands to increase friction (steric hindrance) between the chelates during twist motions; adding steric groups to the C‐backbone of the ligands is anticipated to be less effective.

The calculated Δ‐*mer* and Δ‐*fac* isomeric structures of *N*‐methyl substituted Al(acnac)_3_^Me^ are shown in Figure 7. Introduction of the *N*‐methyl groups enhances the energic preference of the *mer* isomer over the *fac* isomer from 1.7 (*1.7*) to 5.7 *(5.8)* kcal mol^−1^ due to the close proximity of the three methyl groups in the latter. Hence, in an equilibrium situation, the *fac* isomer would not be detectable. The steric impact of *N*‐methyl substitution is far greater on the four racemization pathways. The only ones that remain viable are the enantiomeric *d,l*‐*trans* ^Me^ twists (Ray–Dutt), shown in Figure 7, for which the barriers have increased from 20.2 *(19.9)* kcal mol–^1^ for the unsubstituted Al(acnac)_3_ to a substantial 28.1 *(28.4)* mol^−1^ for Al(acnac)_3_^Me^.

**FIGURE 7:**
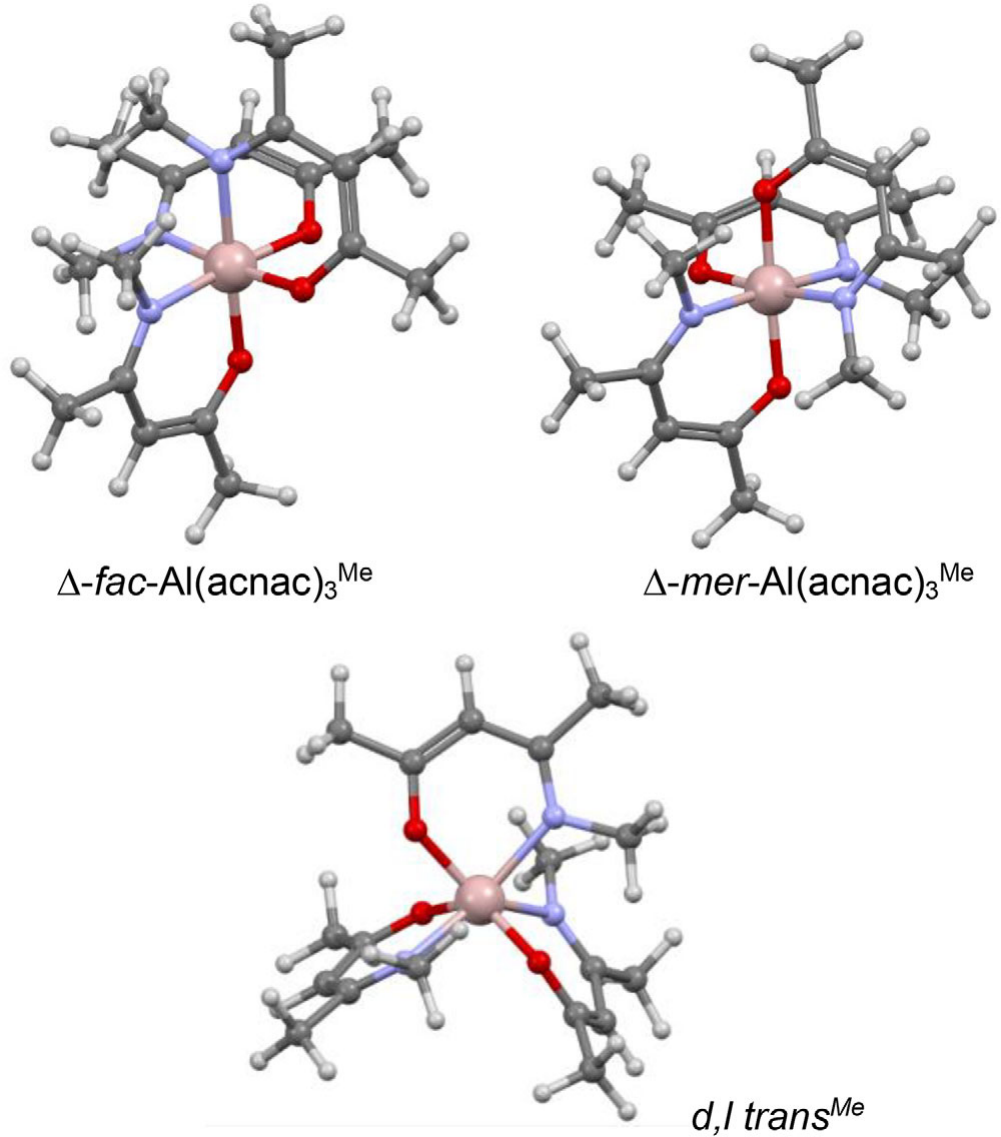
Molecular structures of the Δ‐*fac*‐ and Δ‐*mer*‐Al(acnac)_3_^Me^ isomers, and the *d,l*‐*trans* ^Me^ transition structure for racemization of the methyl‐substituted Al(acnac)_3_ complex.

Geometry optimization of the perceived *cis*, *trans*, and *d,l‐cis* transition structures for Al(acnac)_3_^Me^ yielded only the *mer* and *fac* minima. The reason for this is simply too much steric repulsion between the *N*‐methyl groups in the perceived transition structures as highlighted in Figure 8. As a consequence, both Bailar twist pathways (*cis* and *trans*) are completely inaccessible as the associated H···H contacts become untenable. Likewise, the *mer‐fac* isomerization pathway via the *d,l*‐cis Ray–Dutt twist is equally inaccessible.

**FIGURE 8:**
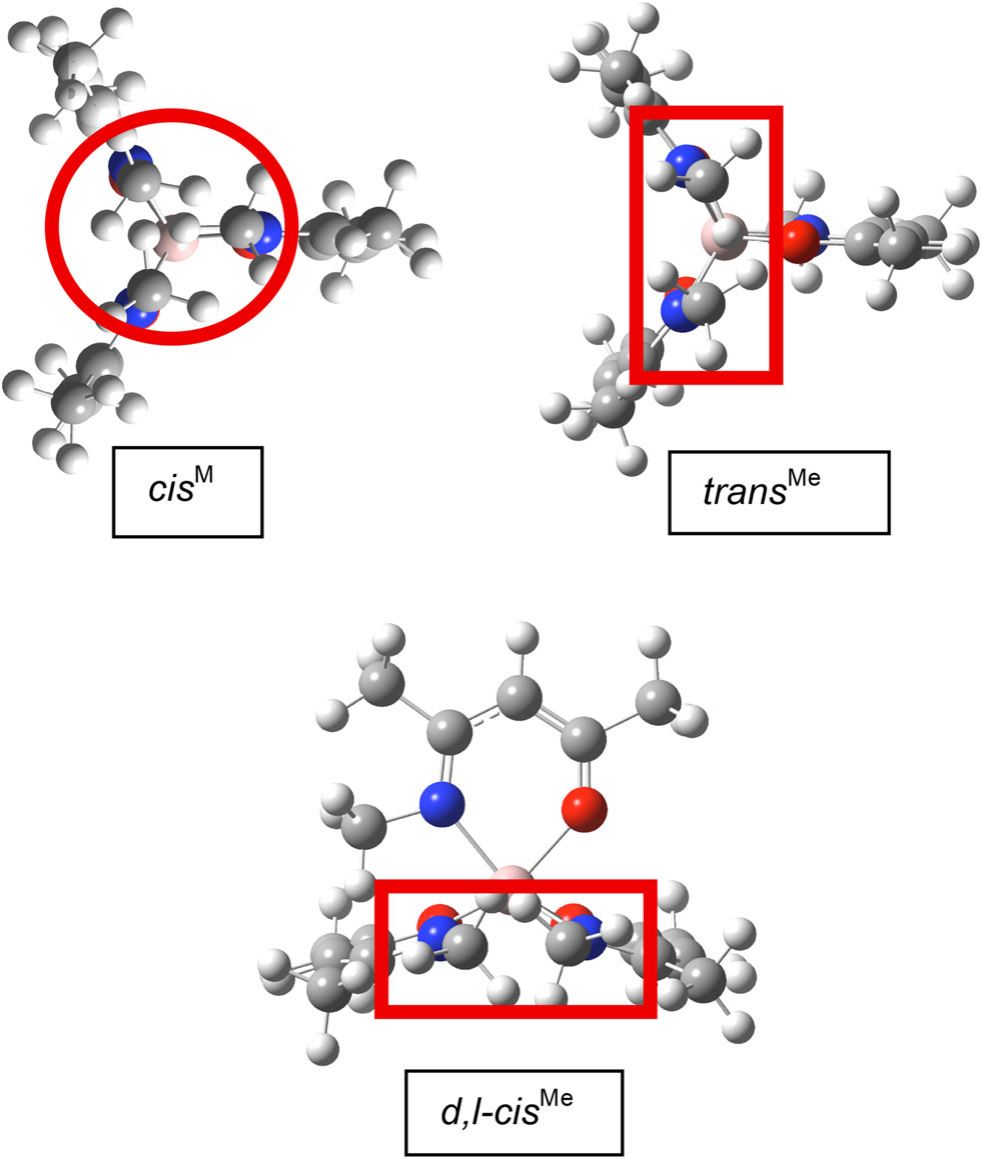
Input geometries for the *cis*, *trans*, and *d,l‐cis* transition structures of Al(acnac)_3_^Me^. The highlighted circle shows clashes between the three N‐Me groups and the highlighted boxes shows these between two of the N‐Me groups.

It then appears that even simple *N*‐methyl substitution of the three acnac ligands of Al(acnac)_3_ induces profound changes in its stereochemical dynamics with major consequences. The *mer* isomer of Al(acnac)_3_^Me^ and its 5.7 *(5.8)* kcal mol^−1^ less stable *fac* isomer can no longer interconvert! Moreover, if a *fac* ^Me^ enantiomer were to be attainable, it would not be able to racemize by means of a *cis* (Bailar) twist. The only remaining racemization pathway for the *mer* global minimum is by means of the *d,l*‐*trans* ^Me^ twists (Ray–Dutt), but these enantiomeric pathways have very high barriers of 28.1 *(28.4)* mol^−1^, suggesting that the *mer* isomer may be separable into its enantiomers. Bulkier N‐substituents are expected to further increase the racemization barrier, thereby enhancing the chiral integrity of the complex.

## 4 Conclusions

The racemization and isomerization mechanisms described in this work are expected to be generic for all octahedral M(a‐chel)_3_ complexes. Methylation of all three N‐sites of the acnac chelate is sufficient to achieve chiral integrity as the 26.0 kcal mol^−1^ calculated Gibbs free energy barrier through the *d,l* trans^Me^ amounts to a rate constant at 25°C; k ≈ 5.2×10^−7^ s^−1^ and a half‐life of 15.4 days. The applicability of this method to a broader pool of transition‐metal complexes should be approached with caution, given the presence of higher‐lying electronic states where racemization occurs. Consideration of the contributions of all possible electronic states and their impact on racemization is underway in our laboratory.

## Conflicts of Interest

The authors declare no conflicts of interest.

## Supporting information




**Supporting File 1**: chem71610‐sup‐0001‐SuppMat.pdf

## Data Availability

The data that support the findings of this study are available in the Supporting Information of this article.
