# Supplementary material for: Chiral‐at‐Metal Racemization of Tris‐Chelated Octahedral Complexes: A DFT Case Study of Al(acnac)3
Source: Chemistry. 2026 Aug 21;32(42):e71610. doi: 10.1002/chem.71610 (PMC13755182; doi:10.1002/chem.71610)
Supplement: Supplementary file 1 [file CHEM-32-e71610-s001.pdf]

Electronic Supporting Information

Chiral-at-Metal Racemization of tris-Chelated Octahedral Complexes. A DFT Case Study of $Al(acnac)_3$

George Dhimba,[a] Alfred Muller,[a] and Koop Lammertsma[a,b,*]

[a] Department of Chemical Sciences, University of Johannesburg, Auckland Park, Johannesburg, 2006, South Africa

[b] Department of Chemistry and Pharmaceutical Sciences, Faculty of Sciences, Vrije Universiteit Amsterdam, De Boelelaan 1108, 1081 HZ Amsterdam, The Netherlands.

Email address: k.lammertsma@vu.nl

ENERGIES

Table S1. Absolute energy terms in a.u and their relative (Δ) in kcal mol^{-1} for geometric *mer* and *fac* isomers of Al(acnac)$_3$, and (*d,l-cis*, *d,l-trans*, *cis*, and *trans*) transition structures calculated at the ωB97XD/def2-TZVPP level.

	***E* (a.u)**	**Δ*E***	***E*$_{ZPVE}$ (a.u)**	**Δ*E*$_{ZPVE}$**	***H* (a.u)**	**Δ*H***	***G*(a.u)**	**Δ*G***
mer	-1218.73765	0.0	-1218.35583	0.0	-1218.32868	0.0	-1218.41455	0.0
fac	-1218.73499	1.7	-1218.35332	1.6	-1218.32610	1.6	-1218.41334	0.8
d,l cis	-1218.69508	26.7	-1218.31381	26.4	-1218.28722	26.0	-1218.37030	27.8
d,l trans	-1218.70538	20.2	-1218.32377	20.1	-1218.29816	19.1	-1218.37891	22.4
cis	-1218.69149	29.0	-1218.31080	28.3	-1218.28375	28.2	-1218.37060	27.6
trans	-1218.70608	19.8	-1218.32477	19.5	-1218.29796	19.3	-1218.38376	19.3

Table S2. Absolute energy terms in a.u and their relative (Δ) in kcal mol^{-1} for geometric *mer* and *fac* isomers of Al(acnac)$_3$, and (*d,l-cis*, *d,l-trans*, *cis*, and *trans*) transition structures calculated at the ωB97XD/ 6-311++G(d,p) level.

	***E* (a.u.)**	**Δ*E***	***E*$_{ZPVE}$ (a.u.)**	**Δ*E*$_{ZPVE}$**	***H* (a.u.)**	**Δ*H***	***G*(a.u.)**	**Δ*G***
Mer	-1218.61601	0.0	-1218.23496	0.0	-1218.20511	0.0	-1218.29191	0.0
Fac	-1218.61329	1.7	-1218.23235	1.6	-1218.20511	0.0	-1218.29191	0.0
d,I cis	-1218.57290	27.1	-1218.19269	26.5	-1218.16594	24.6	-1218.24958	26.6
d,l trans	-1218.58430	19.9	-1218.20360	19.7	-1218.17794	17.0	-1218.25825	21.1
Cis	-1218.56887	29.6	-1218.18929	28.7	-1218.16211	27.0	-1218.24901	26.9
Trans	-1218.58516	19.4	-1218.20478	18.9	-1218.17883	16.5	-1218.26071	19.6

Table S3. Absolute energy terms in a.u. and their relative (Δ) in kcal mol^{-1} for the methylated geometric *mer* and *fac* isomers of Al(acnac)$_3^{Me}$, along with the *d,l–trans^{Me}* twist. All data were obtained at the ωB97XD/def2-TZVPP level of theory.

	E (a.u.)	**Δ*E***	***E$_{ZPVE}$* (a.u.)**	***ΔE$_{ZPVE}$***	***H (a.u.)***	***ΔH***	***G(a.u.)***	***ΔG***
Mer	-1336.65132	0.0	-1336.18421	0.0	-1336.15393	0.0	-1336.24132	0.0
Fac	-1336.64229	5.7	-1336.17494	5.8	-1336.14471	5.8	-1336.23219	5.7
d,l trans	-1336.60655	28.1	-1336.1409	27.2	-1336.11059	27.2	-1336.1998	26.0

Table S4. Absolute energy terms in a.u. and their relative (Δ) in kcal mol^{-1} for the methylated geometric *mer* and *fac* isomers of Al(acnac)$_3^{Me}$, along with the *d,l–trans^{Me}* twist. All data were obtained at the ωB97XD/6-311++G(d,p) level of theory.

	***E* (a.u.)**	**Δ*E***	***E*$_{ZPVE}$ (a.u.)**	**Δ*E*$_{ZPVE}$**	***H* (a.u.)**	**Δ*H***	***G*(a.u.)**	**Δ*G***
Mer	-1336.51808	0.0	-1336.05184	0.0	-1336.021558	0.0	-1336.10892	0.0
Fac	-1336.50880	5.8	-1336.04236	6.0	-1336.01212	5.9	-1336.09960	5.8
d,l trans	-1336.47275	28.4	-1336.0078	27.6	-1335.97764	27.6	-1336.0664	26.7

Intrinsic reaction coordinate (IRC) scans

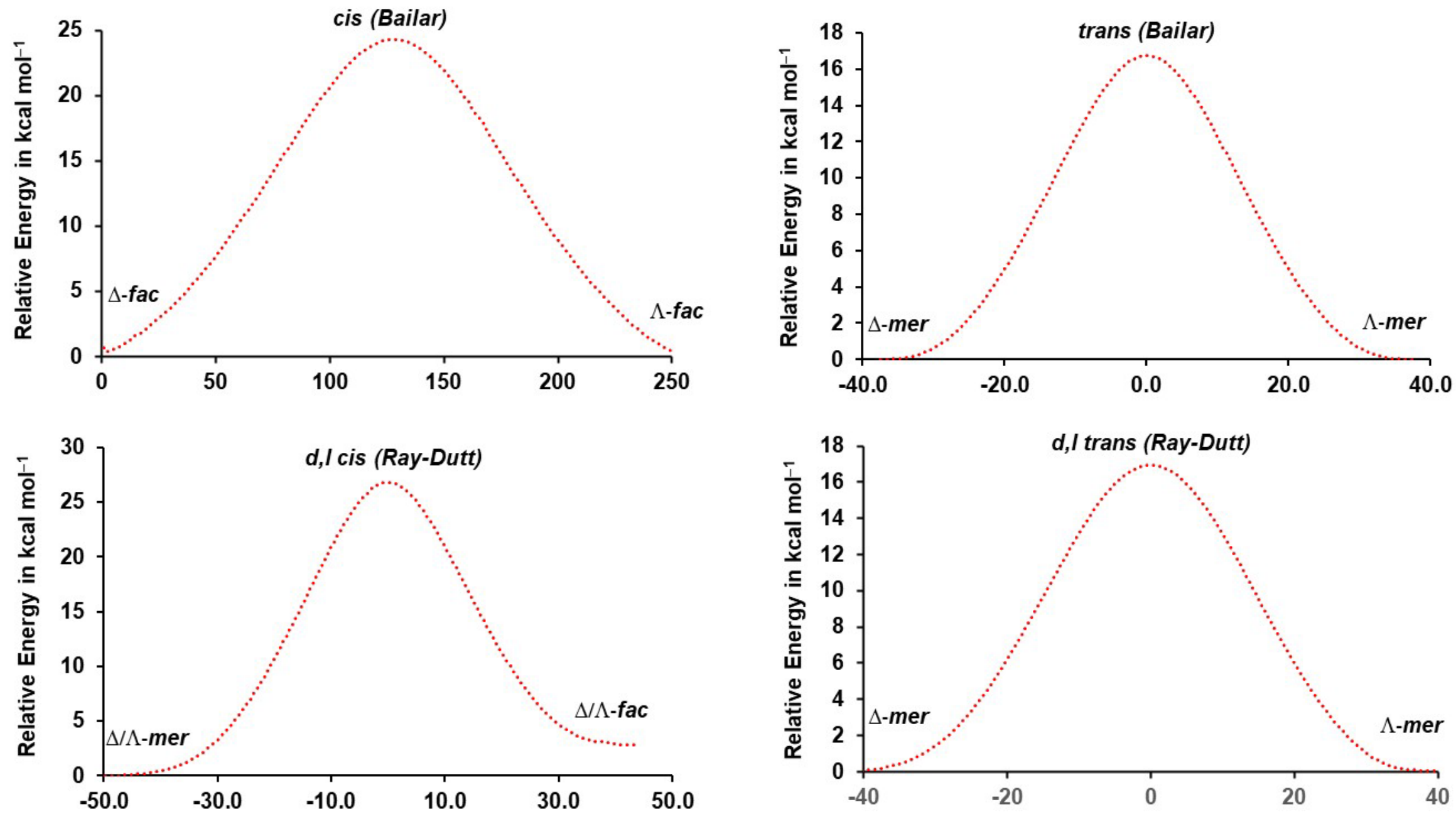

Figure S1. IRC) scans for the racemisation and isomerisation pathways of Al(acnac)$_3$, calculated at the ωB97X-D/6-31G(d) level.

Input geometries for methylated Bailar (cis and trans) and Ray-Dutt (d,l cis) twists

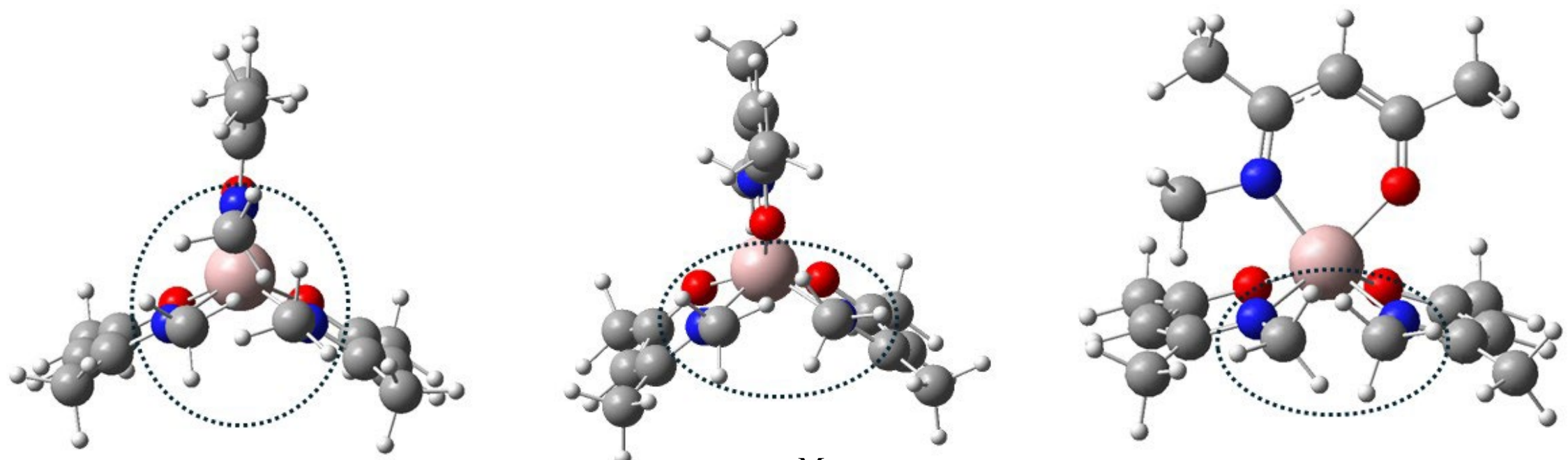

cis^{Me} trans^{Me} d,l cis^{Me}

Figure S2. Input structures for the *cis*, *trans*, and *d,l-cis* transition structures of Al(acnac)$_3^{Me}$.

Generated using GaussView input geometries *cis*^{Me}, *trans*^{Me}, and *d,l trans*^{Me} for the Al(acnac)$_3^{Me,}$ rearrangement shows significant steric hindrance caused by added methyl groups. This renders the three transition structures unviable, and their associated reactive channels are inhibited.

Geometries

Δ-*fac*-Al(acnac)$_3$

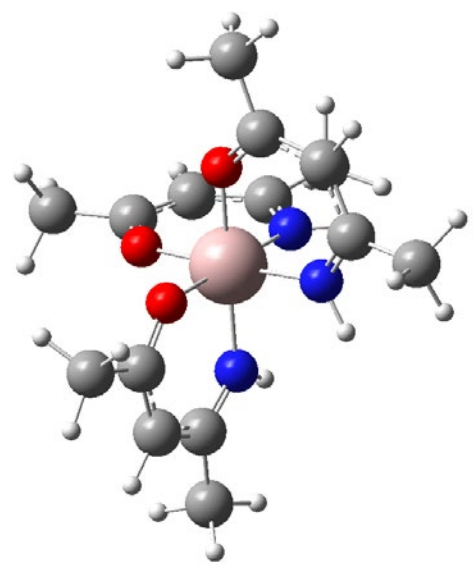

Atom	X	Y	Z
C	-1.040483	2.499472	1.000196
C	-0.479461	3.298701	0.015672
H	-0.641910	4.364938	0.060253
C	0.340052	2.794299	-1.014976
C	-1.655022	-2.147589	0.999840
C	-2.624810	-2.059212	0.012560
H	-3.470970	-2.727967	0.057479
C	-2.588488	-1.102839	-1.022760
H	-1.739491	0.381554	-1.937076
O	-0.621862	-1.415254	1.069485
N	-1.597389	-0.267544	-1.175741
N	0.575193	1.519738	-1.167902
H	1.214774	1.322231	-1.924927
O	-0.915135	1.239297	1.070316
C	-1.790545	-3.166755	2.097220
H	-1.823011	-2.649195	3.056631
H	-2.680335	-3.782943	1.988495
H	-0.905488	-3.803860	2.103693
C	-3.770777	-1.042680	-1.953054
H	-4.087456	-2.043850	-2.242975
H	-4.610282	-0.568904	-1.441588
H	-3.546869	-0.468663	-2.851478
C	0.983818	3.792740	-1.939911
H	1.799931	4.295843	-1.418750
H	1.387683	3.313814	-2.831129
H	0.269068	4.557160	-2.242570
C	-1.864176	3.121048	2.094074
H	-1.959037	4.199206	1.985337
H	-2.855710	2.667019	2.095763
H	-1.402799	2.892861	3.055554
Al	0.001113	-0.002398	-0.013059
O	1.530584	0.165126	1.076037
C	2.257877	-1.687700	-1.016463
C	3.101571	-1.233219	0.017898
H	4.106767	-1.624105	0.062957
C	2.685819	-0.353722	1.006034
N	1.036090	-1.255407	-1.169885
H	0.547253	-1.708296	-1.929540
C	3.632193	0.044188	2.104837
H	4.614711	-0.409665	1.995633
H	3.732028	1.130102	2.113821
H	3.202210	-0.248587	3.063375
C	2.804304	-2.739552	-1.944842
H	2.831530	-3.700286	-1.428113
H	2.190189	-2.846221	-2.838283
H	3.824280	-2.500279	-2.243524

Δ-*mer*-Al(acnac)$_3$

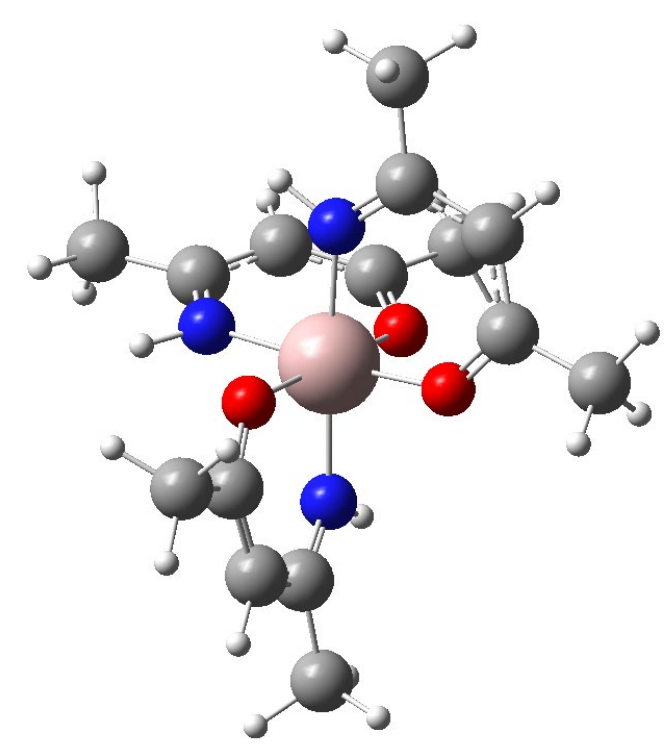

Atom	X	Y	Z
O	-1.424160	-0.040214	-1.308480
O	-0.685321	1.418563	0.906014
C	-3.243410	-0.986152	-0.151341
H	-4.303468	-1.188636	-0.180679
C	0.578130	3.171223	-0.024459
H	0.782233	4.230838	0.007597
C	-3.272173	-2.182338	2.036768
H	-3.990061	-1.511127	2.510348
H	-2.609423	-2.578954	2.804440
H	-3.836021	-3.004756	1.596478
C	-1.070654	3.542866	1.820615
H	-2.145699	3.461171	1.655900
H	-0.768685	4.583816	1.728069
H	-0.871050	3.197007	2.835592

C	2.214047	3.105871	-1.902253
H	1.660069	3.796587	-2.539314
H	2.763138	2.410897	-2.536044
H	2.927826	3.695038	-1.326361
C	-3.504107	0.100137	-2.388569
H	-3.098106	-0.337575	-3.301462
H	-3.439426	1.183910	-2.493951
H	-4.547157	-0.189721	-2.282328
C	-2.511133	-1.436544	0.974557
C	-2.664613	-0.326423	-1.216224
C	1.264221	2.389526	-0.980218
C	-0.355730	2.645875	0.848465
C	2.333568	-3.330171	-1.738177
C	1.894276	-2.213379	-0.829423
C	2.782382	-1.829642	0.202916
C	2.434616	-0.949364	1.213332
C	3.402277	-0.663514	2.328486
H	3.326980	-3.125205	-2.137541
H	2.396748	-4.262030	-1.174709
H	1.642932	-3.470455	-2.568455
H	3.749092	-2.308613	0.244159
H	2.933735	-0.910967	3.281752
H	4.332543	-1.218307	2.228246
H	3.617450	0.405679	2.343775
Al	-0.049982	-0.100606	-0.006311
N	1.091597	1.104046	-1.090626
H	1.648376	0.691736	-1.825630
N	-1.237843	-1.218733	1.110817
H	-0.851231	-1.593756	1.965367
O	1.317159	-0.347177	1.299394
N	0.731505	-1.651647	-0.992750
H	0.191104	-2.048318	-1.748725

***trans* (Bailar twist)**

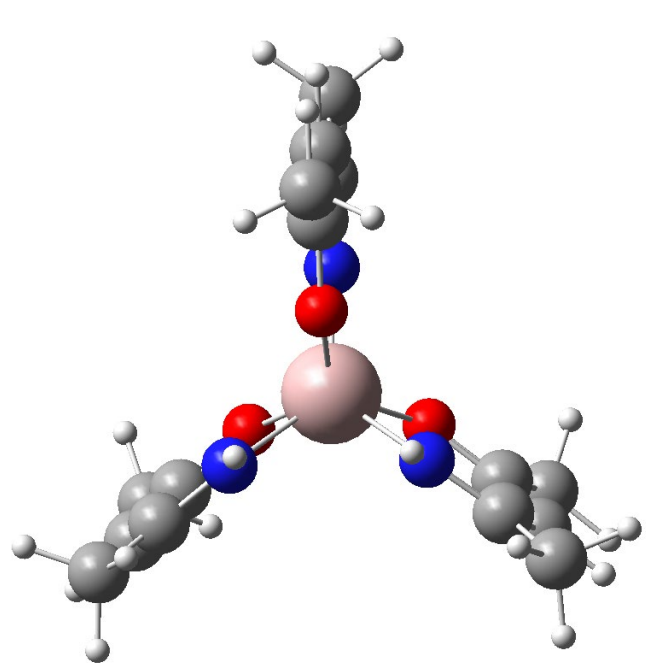

Atom	X	Y	Z
O	-1.69133	0.009535	-1.16957
O	0.82266	-1.25229	1.207149
C	-2.73314	0.009261	1.462922
C	-3.51991	0.015184	0.291964
H	-4.59491	0.019986	0.387458
C	-2.94592	0.014955	-0.962706
C	1.61157	-2.23548	1.064881
C	1.907799	-2.81561	-0.154358
H	2.582231	-3.65687	-0.199457
C	1.268201	-2.38618	-1.332152
N	-1.43241	0.003295	1.431175
N	0.456685	-1.36502	-1.350284
H	-1.0057	-0.00046	2.347248
H	0.03136	-1.22238	-2.255733
C	-3.80885	0.021357	-2.195763
H	-3.57994	-0.85793	-2.799578
H	-4.87077	0.025798	-1.959457
H	-3.57182	0.900856	-2.796128
C	-3.45218	0.010001	2.784785
H	-2.75739	0.005327	3.623000
H	-4.08965	0.891345	2.862034
H	-4.09748	-0.86591	2.858737
C	2.213555	-2.77316	2.334333
H	2.885677	-3.60946	2.153856
H	2.756714	-1.97358	2.839236
H	1.412305	-3.0933	3.001695
C	1.523096	-3.16893	-2.592433
H	2.594452	-3.23201	-2.784405
H	1.152056	-4.18823	-2.479417
H	1.03945	-2.71475	-3.456161
O	0.834199	1.238809	1.211970
C	1.290273	2.378364	-1.322916
C	1.933855	2.797228	-0.143486
H	2.616088	3.63235	-0.185341
C	1.63224	2.21515	1.073497
N	0.469278	1.364889	-1.344985
H	0.042628	1.22974	-2.250960
C	2.239208	2.742252	2.345010
H	2.919087	3.572958	2.167767
H	1.440977	3.067234	3.013648
H	2.774903	1.935687	2.846771
C	1.552544	3.163535	-2.580172

H	1.063892	2.717796	-3.445475
H	1.192034	4.186129	-2.462900
H	2.624407	3.216393	-2.772420
Al	-0.07755	-0.00022	0.011967

cis (Bailar twist)

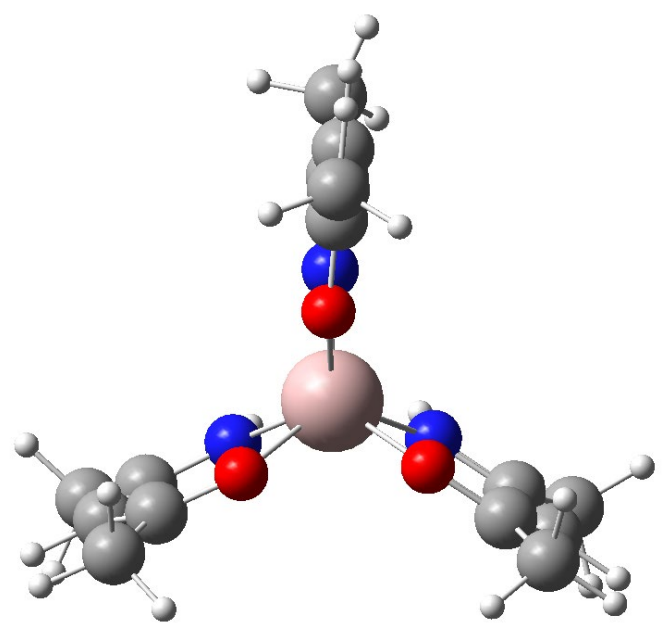

Atom	X	Y	Z
O	-0.785579	1.276883	1.164536
C	2.571535	-0.083449	1.42949
C	3.437097	-0.036564	0.350723
H	4.503315	-0.029500	0.515803
C	2.945244	-0.027294	-0.96411
C	-1.446173	2.354192	1.116252
C	-1.741684	3.026448	-0.0579
H	-2.304357	3.946286	-0.02415
C	-1.288726	2.542139	-1.29408
N	-0.600883	1.433797	-1.40335
H	-0.351940	1.268302	-2.36973
C	3.946172	-0.061611	-2.08908
H	4.795014	0.585283	-1.8722
H	4.328197	-1.077234	-2.20608
H	3.501600	0.249218	-3.03438
C	3.100890	-0.127506	2.836218
H	2.732708	-1.029370	3.326329
H	4.188213	-0.113325	2.872103
H	2.706904	0.724612	3.390901
C	-1.901084	2.894220	2.44387
H	-2.463939	3.820107	2.345535
H	-2.517472	2.143936	2.93996
H	-1.029007	3.064472	3.076018
C	-1.641114	3.322151	-2.5335
H	-1.669709	4.391328	-2.33125
H	-0.929208	3.136391	-3.33791

H	-2.632325	3.023033	-2.87954
O	-0.920795	-1.228233	1.086208
C	-1.361646	-2.402062	-1.42941
C	-1.768031	-2.976579	-0.21554
H	-2.296409	-3.917145	-0.22652
C	-1.524626	-2.335078	0.987156
N	-0.718815	-1.263380	-1.48565
H	-0.533290	-1.011487	-2.44761
C	-1.991489	-2.935555	2.284377
H	-2.501183	-3.886351	2.143054
H	-1.131895	-3.076752	2.940192
H	-2.663008	-2.233422	2.779574
C	-1.659654	-3.152197	-2.70111
H	-0.894613	-3.914516	-2.85968
H	-2.622008	-3.657775	-2.64183
H	-1.664636	-2.488783	-3.56601
Al	-0.012422	0.016279	-0.04799
O	1.310762	-0.098109	1.322624
N	1.664956	-0.02305	-1.23266
H	1.515572	-0.03236	-2.23306

d,l trans (Ray-Dutt twist)

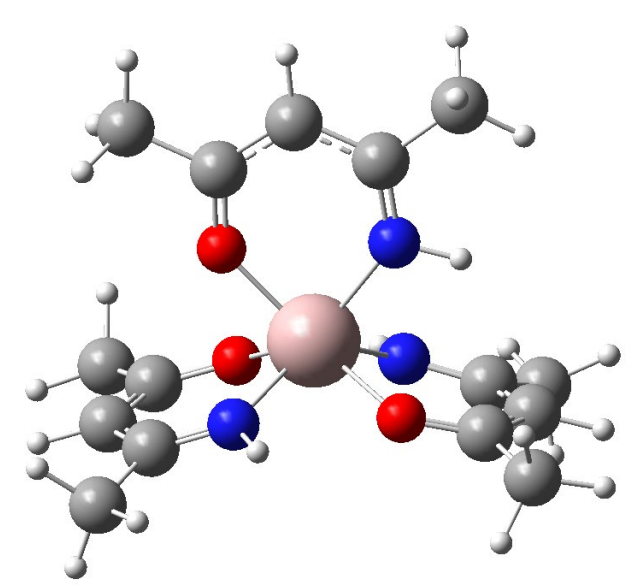

Atom	X	Y	Z
C	2.536923	-1.216352	-1.278092
C	3.250904	-1.185638	-0.057093
H	4.301969	-1.431789	-0.074878
C	2.615454	-1.047801	1.167920
C	-3.009470	-1.538011	0.102080
H	-4.033395	-1.878994	0.131341
C	-2.310645	-1.416694	1.322066
H	0.882146	-1.055198	-2.278588
H	-0.686851	-1.016779	2.313847

O	-1.188983	-0.996365	-1.280088
O	1.381276	-0.792792	1.306150
N	1.277982	-0.899738	-1.362306
N	-1.092189	-0.961991	1.391117
C	-2.402660	-1.349878	-1.123882
C	-3.168045	-1.597386	-2.393941
H	-4.183605	-1.940602	-2.208924
H	-3.200709	-0.672353	-2.971652
H	-2.640329	-2.338589	-2.995451
C	-3.018458	-1.861867	2.573160
H	-2.388260	-1.753698	3.454583
H	-3.920803	-1.265784	2.715966
H	-3.327205	-2.903618	2.484632
C	3.280527	-1.682634	-2.500995
H	4.227380	-1.150486	-2.593572
H	3.511910	-2.744853	-2.410779
H	2.700297	-1.526843	-3.409123
C	3.393606	-1.225658	2.444328
H	2.962691	-2.052432	3.010588
H	4.448091	-1.425880	2.266716
H	3.292778	-0.327293	3.054644
O	-1.370365	1.222965	-0.101012
C	0.975527	2.761453	0.015936
C	-0.320666	3.308730	-0.012848
H	-0.440549	4.381177	0.001468
C	-1.431640	2.489763	-0.073041
N	1.188131	1.473930	0.011615
H	2.176083	1.252647	0.027181
C	-2.817749	3.071949	-0.114308
H	-2.813198	4.159536	-0.082152
H	-3.318298	2.738709	-1.024243
H	-3.391974	2.686353	0.728936
C	2.139732	3.715320	0.052142
H	3.093173	3.188845	0.064417
H	2.113229	4.372772	-0.817350
H	2.077677	4.346603	0.939193
Al	0.006914	-0.143432	-0.019018

d,l cis **(Ray-Dutt twist)**

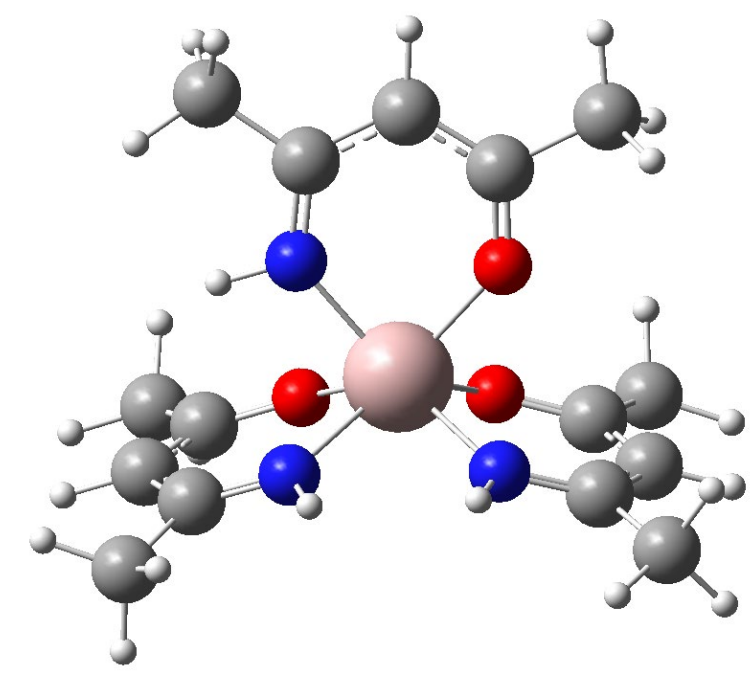

Atom	X	Y	Z
C	-2.304065	-1.256798	1.330162
C	-2.988502	-1.620072	0.183164
H	-3.995708	-1.997691	0.271444
C	-2.395508	-1.585192	-1.090371
C	2.534865	-0.948288	1.313042
C	3.273021	-1.187093	0.163474
H	4.319053	-1.437628	0.254837
C	2.672698	-1.267496	-1.111676
H	1.168703	-1.114589	-2.285209
O	1.297957	-0.678777	1.325383
N	1.422299	-0.960630	-1.318931
N	-1.186827	-1.131380	-1.298948
H	-0.928627	-1.221129	-2.271196
O	-1.107612	-0.837010	1.349327
C	3.193702	-1.019784	2.663438
H	2.692065	-1.781413	3.261207
H	4.254787	-1.251196	2.599243
H	3.058791	-0.067225	3.177090
C	3.529591	-1.764817	-2.246195
H	4.493290	-1.255925	-2.248160
H	3.724535	-2.830850	-2.120395
H	3.050993	-1.610908	-3.212758
C	-3.212457	-2.081660	-2.253569
H	-3.916598	-1.303501	-2.554212
H	-2.585675	-2.323689	-3.111657
H	-3.790743	-2.962919	-1.980763
C	-2.963974	-1.372874	2.675730
H	-3.969092	-1.784406	2.613749
H	-2.351200	-2.001679	3.322130
H	-3.006192	-0.383307	3.133021
Al	0.013748	-0.148635	-0.014248

O	-1.391448	1.204126	-0.140388
C	0.938674	2.765583	-0.226664
C	-0.362125	3.299198	-0.241307
H	-0.493256	4.369312	-0.288493
C	-1.466212	2.468374	-0.194442
N	1.164885	1.480567	-0.170524
H	2.155375	1.270352	-0.166281
C	-2.859003	3.036428	-0.209580
H	-2.864627	4.123257	-0.261471
H	-3.402330	2.630142	-1.063578
H	-3.384714	2.714009	0.689957
C	2.096085	3.727998	-0.255546
H	2.167650	4.241604	0.704515
H	3.039753	3.217456	-0.443976
H	1.945917	4.487651	-1.022089

Mer-Al(acnac)$_3^{Me}$

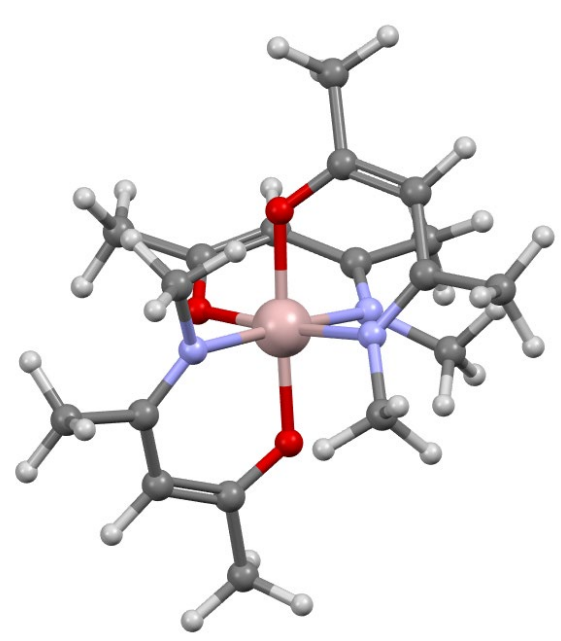

Atom	X	Y	Z
**O**	-1.423956	-0.192498	-1.467222
O	-0.862573	1.305165	0.749503
C	-3.337582	-0.744759	-0.225791
H	-4.416401	-0.738797	-0.219213
C	0.911611	2.792242	0.340728
H	1.365174	3.724008	0.642143
C	-3.563805	-2.015458	1.900941
H	-3.278697	-1.727275	2.912600
H	-3.483036	-3.102270	1.835496
H	-4.604043	-1.746493	1.739383
C	-0.795011	3.209017	2.121726
H	-1.838099	3.433202	1.895463
H	-0.249564	4.137917	2.273420
H	-0.781066	2.627460	3.044802
C	2.436808	2.940990	-1.621986
H	1.994573	3.354204	-2.530710
H	3.263650	2.298002	-1.924224
H	2.828778	3.767847	-1.035594
C	-3.478460	0.269471	-2.507792
H	-3.176764	-0.271332	-3.405953
H	-3.232797	1.320740	-2.664633
H	-4.552448	0.167422	-2.369346
C	-2.676690	-1.352607	0.877486
C	-2.695071	-0.244256	-1.334111
C	1.406778	2.170520	-0.835839
C	-0.219546	2.374539	1.012461
C	2.656407	-3.210181	-1.581165
C	2.018349	-2.104653	-0.780297
C	2.876604	-1.363324	0.074061
C	2.417544	-0.514155	1.060642
C	3.368319	0.117156	2.035768
H	3.740041	-3.148585	-1.528746
H	2.355770	-4.186090	-1.195228
H	2.349117	-3.166746	-2.626426
H	3.934666	-1.569865	0.027975
H	3.078148	-0.159375	3.050216
H	4.402191	-0.173588	1.862934
H	3.273050	1.201564	1.957129
Al	-0.150032	-0.208620	-0.098120
N	0.976082	1.013498	-1.256956
N	-1.379371	-1.373714	1.001747
O	1.189415	-0.221813	1.233709
N	0.736962	-1.869141	-0.868213
C	1.388791	0.550312	-2.569573
H	1.385245	1.348856	-3.312950
H	2.390668	0.109268	-2.541974
H	0.691028	-0.212139	-2.905430
C	-0.084934	-2.772455	-1.657556
H	-1.124758	-2.645254	-1.371110
H	-0.011364	-2.553611	-2.727521
H	0.183576	-3.818829	-1.504186
C	-0.790546	-2.131639	2.094015
H	0.233232	-2.385251	1.833814
H	-0.754952	-1.538662	3.011865
H	-1.329190	-3.057494	2.298550

Fac-Al(acnac)$_3^{Me}$

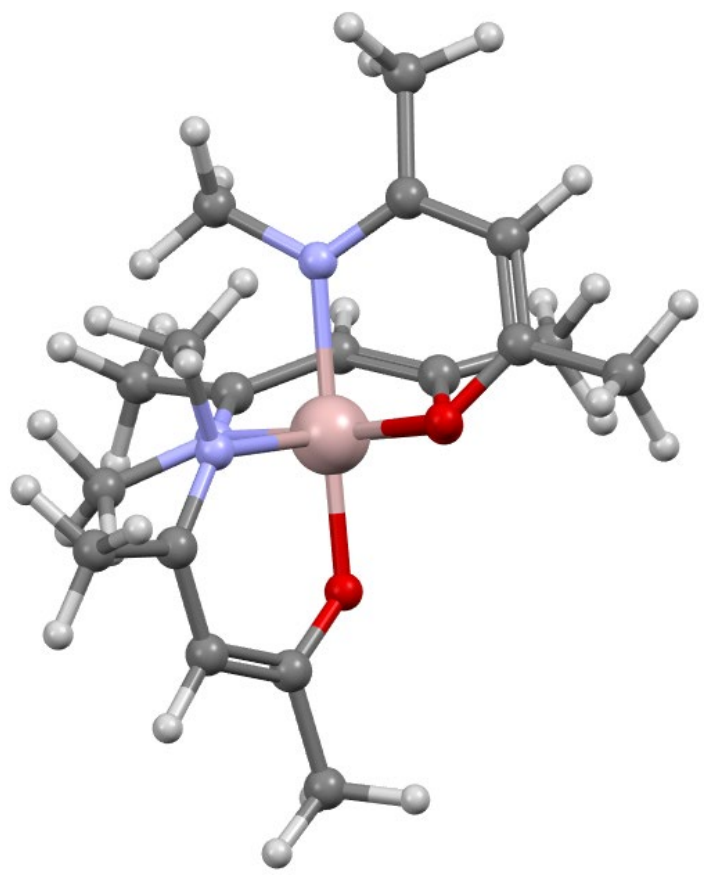

Atom	X	Y	Z
C	-2.07470	1.14883	1.39785
C	-2.01014	2.26797	0.58703
H	-2.83872	2.95892	0.60559
C	-0.84645	2.65505	-0.12110
C	-1.08197	-2.58008	0.69731
C	-2.21363	-2.25494	-0.02784
H	-3.08223	-2.88647	0.07715
C	-2.26185	-1.23259	-1.00512
O	0.00295	-1.91618	0.66751
N	-1.28110	-0.38757	-1.20564
N	0.19626	1.87775	-0.27061
O	-1.15503	0.28005	1.48303
C	-1.06760	-3.79679	1.57783
H	-0.89465	-3.48115	2.60764
H	-1.99428	-4.36388	1.52411
H	-0.23245	-4.43662	1.29083
C	-3.50238	-1.17903	-1.85918
H	-4.27533	-1.82845	-1.45759
H	-3.89129	-0.16202	-1.91776
H	-3.28539	-1.50354	-2.87881
C	-0.85671	4.04836	-0.69921
H	-0.18979	4.70438	-0.13683
H	-0.51741	4.05065	-1.73558
H	-1.85600	4.47292	-0.65574
C	-3.27294	0.90546	2.27081
H	-4.03007	1.68025	2.17131
H	-3.70240	-0.06335	2.01006
H	-2.94916	0.84608	3.31053
Al	0.18198	-0.10394	0.21156
O	1.46052	0.19059	1.53437
C	2.97097	-0.59682	-0.80755
C	3.49657	-0.12324	0.41887
H	4.56973	-0.09001	0.52200
C	2.72877	0.19344	1.52242
N	1.69091	-0.65374	-1.08410
C	3.38200	0.56667	2.82313
H	4.46787	0.56487	2.76056
H	3.03621	1.55559	3.12627
H	3.06217	-0.13668	3.59279
C	3.99713	-1.06572	-1.81041
H	3.98805	-2.15472	-1.88605
H	3.79614	-0.66939	-2.80546
H	4.99469	-0.76081	-1.50687
C	1.32244	-1.31515	-2.33022
H	1.91190	-2.21323	-2.51405
H	0.28288	-1.61654	-2.27736
H	1.44066	-0.65067	-3.19252
C	-1.37016	0.48877	-2.36140
H	-1.64199	-0.05220	-3.27035
H	-2.09698	1.29186	-2.20831
H	-0.40274	0.95267	-2.53335
C	1.38466	2.44138	-0.89166
H	1.32134	2.41584	-1.98596
H	1.56421	3.47406	-0.59218
H	2.25490	1.86272	-0.59986

d,l trans^{Me}

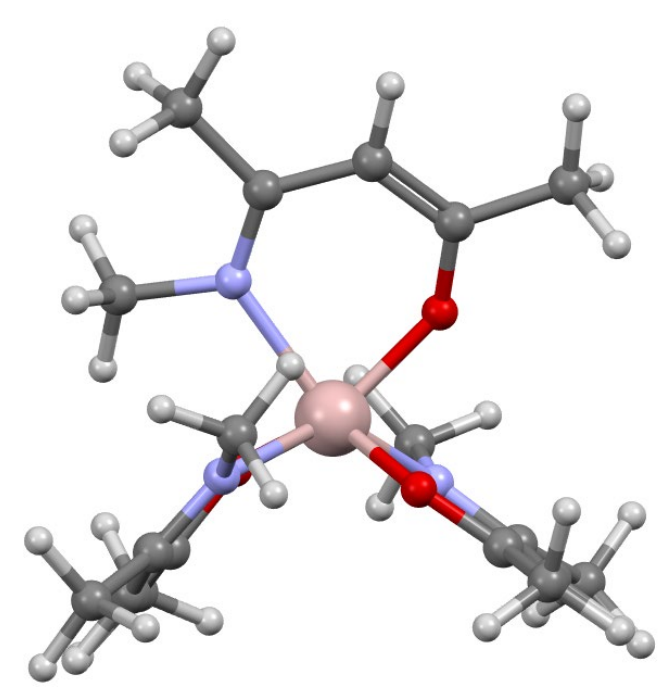

Atom	X	Y	Z
C	2.32508	-1.47389	-1.29047
C	2.68555	-2.09173	-0.05174
H	3.37730	-2.91916	-0.08091
C	2.25343	-1.64549	1.16818
C	-2.56301	-1.96197	0.19876
H	-3.42814	-2.60353	0.13271
C	-2.07148	-1.64539	1.50102
O	-0.99088	-0.72995	-1.03720
O	1.44755	-0.65743	1.33107
N	1.45083	-0.52175	-1.35145
N	-1.04302	-0.88212	1.69660
C	-2.03386	-1.47106	-0.96368
C	-2.65882	-1.79862	-2.29117
H	-3.54029	-2.42824	-2.19149
H	-2.93463	-0.87133	-2.79577
H	-1.92561	-2.30501	-2.92108
C	-2.84583	-2.22264	2.66063
H	-2.18155	-2.67158	3.39901
H	-3.41079	-1.43784	3.16801
H	-3.54923	-2.97743	2.31918
C	3.03311	-1.96288	-2.52743
H	3.66006	-1.17330	-2.94640
H	3.66619	-2.81737	-2.30441
H	2.31776	-2.24830	-3.29962
C	2.73388	-2.28090	2.44097
H	1.88024	-2.70126	2.97559
H	3.46475	-3.06649	2.26272
H	3.17294	-1.51604	3.08299
O	-1.19301	1.35663	0.49915
C	0.94413	2.97718	-0.39924
C	-0.42689	3.32987	-0.47446
H	-0.68755	4.30623	-0.85156
C	-1.41445	2.52244	0.03073
N	1.35833	1.80728	0.01696
C	-2.84434	2.97665	0.09108
H	-2.98145	3.97228	-0.32550
H	-3.46877	2.26476	-0.45015
H	-3.17633	2.97218	1.13025
C	1.92272	4.04867	-0.81156
H	2.61707	3.67926	-1.56702
H	1.40080	4.91354	-1.21150
H	2.52121	4.37509	0.04035
Al	0.14400	0.06286	0.21335
C	2.80429	1.63610	0.10492
H	3.03167	0.68100	0.55759
H	3.27857	1.65796	-0.88092
H	3.26480	2.41502	0.71635
C	-0.71279	-0.51672	3.06579
H	-0.23518	-1.34042	3.60458
H	-0.00910	0.31059	3.06047
H	-1.59455	-0.20444	3.62876
C	1.18305	0.10346	-2.63454
H	0.69373	-0.58523	-3.32697
H	0.51242	0.94797	-2.49142
H	2.09728	0.47552	-3.10566
